# Orogastric tube insertion using the new gastric tube guide: first experiences from a manikin study

**DOI:** 10.1186/s12871-017-0343-1

**Published:** 2017-04-04

**Authors:** Christian Alflen, Marc Kriege, Irene Schmidtmann, Rüdiger R. Noppens, Tim Piepho

**Affiliations:** 1 Department of Anesthesiology, University Medical Center of the Johannes Gutenberg-University Mainz, Langenbeckstrasse 1, Mainz, 55131 Germany; 2 Institute of Medical Biostatistics, Epidemiology, and Informatics, University Medical Center of the Johannes Gutenberg-University Mainz, Langenbeckstrasse 1, Mainz, 55131 Germany; 3 Department of Anesthesia & Perioperative Medicine, Western University, London, ON Canada

**Keywords:** Gastric tube guide, Orogastric tube, Nasogastric tube

## Abstract

**Background:**

Orogastric tube placement is a common procedure routinely used in clinical anesthesiology and intensive care medicine. Nevertheless high failure rates and severe complications have been reported. We conducted this study to evaluate if the usage of the new gastric tube guide would speed up the placement of orogastric tubes and ease the procedure.

**Methods:**

Thirty one professionals were given a hands-on-training in orogastric tube placement in a simulation manikin without and with the gastric tube guide. Afterwards they performed both methods in randomized order. We recorded the placement time, counted the required attempts and asked the participants to rate their experience with both methods.

**Results:**

The median placement time using the gastric tube guide was 14 s compared to 25 s without the device. In addition all participants were able to place the orogastric tube when using the gastric tube guide compared to 26/31 (84%) without it. Furthermore 26/31 (84%) users preferred the gastric tube guide over the standard method.

**Conclusion:**

Our results show that using the gastric tube guide to place orogastric tubes in a manikin led to a significant shorter placement time and a higher overall success rate.

## Background

Insertion of an orogastric tube is a common procedure in clinical anesthesiology to achieve gastric decompression and to reduce gastric contents. The procedure is usually performed after induction of general anaesthesia and tracheal intubation. As the patient is unable to swallow and cannot follow further instructions placement of nasogastric and orogastric tubes can be difficult. This leads to a high failure rate of nearly 50% for the first attempt [1]. As the gastric tube will lose stiffness after warming to body temperature further attempts to place the tube will often result in subsequent failures [2]. Several methods to improve insertion of naso- and orogastric tubes have been published, including the use of different forceps, various head positions, the use of a guidewire to increase the stiffness of the tube and recently the use of a slit endotracheal tube [3]. Further studies showed that the most common sites of resistance at the laryngeal level are the arytenoid cartilages and piriform sinuses [4]. Although the insertion of an orogastric tube is a common procedure several severe complications, like laryngeal injuries [5] and esophageal perforation [6], have been reported.

**Fig. 1:**
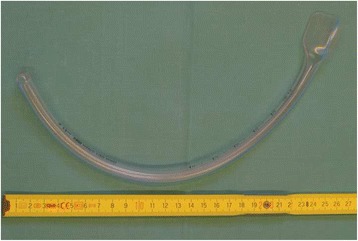
Gastric tube guide - the gastric tube guide is 33 cm in length and has an inner diameter of 7.5 MM

Based on the concept of slit endotracheal tubes the new gastric tube guide (VBM Medizintechnik GmbH, Sulz, Germany) was designed to facilitate the insertion of orogastric tubes. The device is made of polyvinyl chloride, is 33 cm in length, has an outer diameter of 10.3 mm and an inner diameter of 7.5 mm which allows the placement of gastric tubes up to 6.0 mm (18 Fr). According to the manufacturer the gastric tube guide is fitted with an insertion funnel on proximal end to ease the placement of gastric tubes and an atraumatic tip to decrease the risk of mucosal bleeding during the placement of orogastric tubes. It has a precurved shape (Fig. 1) to follow the anatomical structures of the pharynx and is equipped with graduation marks to adjust placement of the device. As slit endotracheal tubes the gastric tube guide has a slit shaft to allow the removal of the device without removing the orogastric tube.

The design of this study was made to investigate if the usage of the gastric tube guide leads to a higher success rate and a faster placement of orogastric tubes in a simulation manikin.

## Methods

**Fig. 2:**
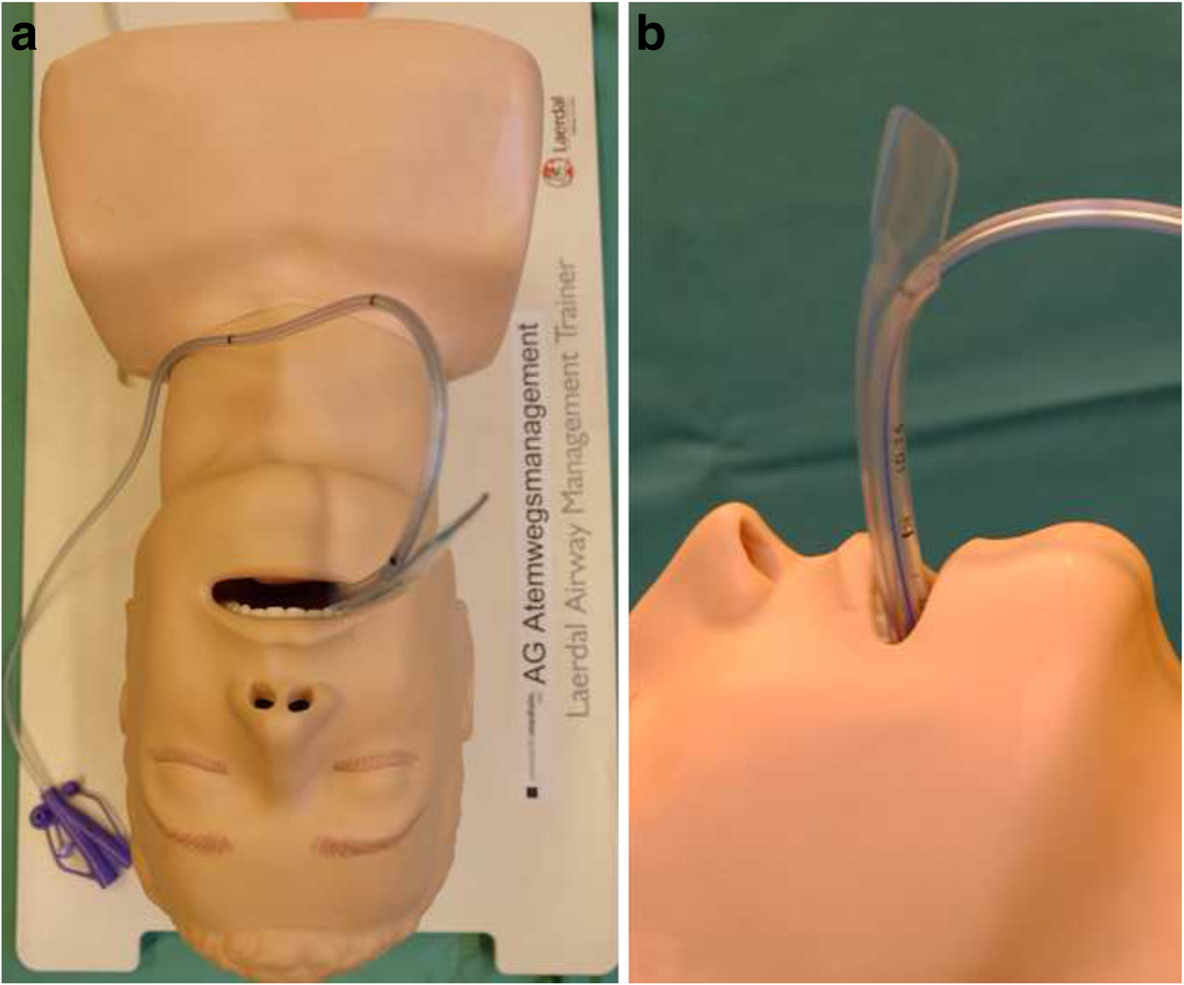
Manikin with the gastric tube guide – (**a**) Experimental set-up with the manikin and an orogastric tube placed through a gastric tube guide; (**b**) Detail of the gastric tube guide with graduation marks (cm)

The Ethics committee of the medical association of Rhineland-Palatinate did not require a formal approval of the study. Thirty one professionals of the Department of Anesthesiology participated in this study, including fourteen nurses, ten residents and seven specialists. Each participant was given a hands-on demonstration for the placement of the orogastric tube with and without the gastric tube guide (Fig. 2). The participants had to use both methods in randomized order, randomization was performed using the Research Randomizer Software [7]. The simulation manikin (“Airway Management Trainer”, Laerdal Medical GmbH, Puchheim, Germany) and gastric tube (14 Fr “Salem Sump PVC Gastroduodenal Tube”, Covidien Deutschland GmbH, Neustadt/Donau, Germany) were the same for all participants and no further tools were allowed. We recorded the times required for successful gastric intubation and the number of attempts. Each complete removal of the tube from the manikin was counted as a new attempt. After the procedure the participant was asked to rate his or her overall experience with the tool ranging from 1 (best) to 6 (worst).

### Statistical analysis

Descriptive analysis of the time to successful placement of the orogastric tube with each method was performed using Kaplan-Meier estimates. Treatment effects and period effects were analyzed using Cox regression including a shared frailty term to take random participant effects into account. To describe the number of attempts and rating, absolute and relative frequencies are reported. The proportion of overall success are compared using the McNemar test, the preference and the number of attempts are evaluated using the Bowker test; *p* values less than 0.05 were considered as significant.

Statistical analyses were performed using SAS 9.4 (2002–2012 SAS Institute Inc., Cary, NC, USA.)

## Results

**Fig. 3:**
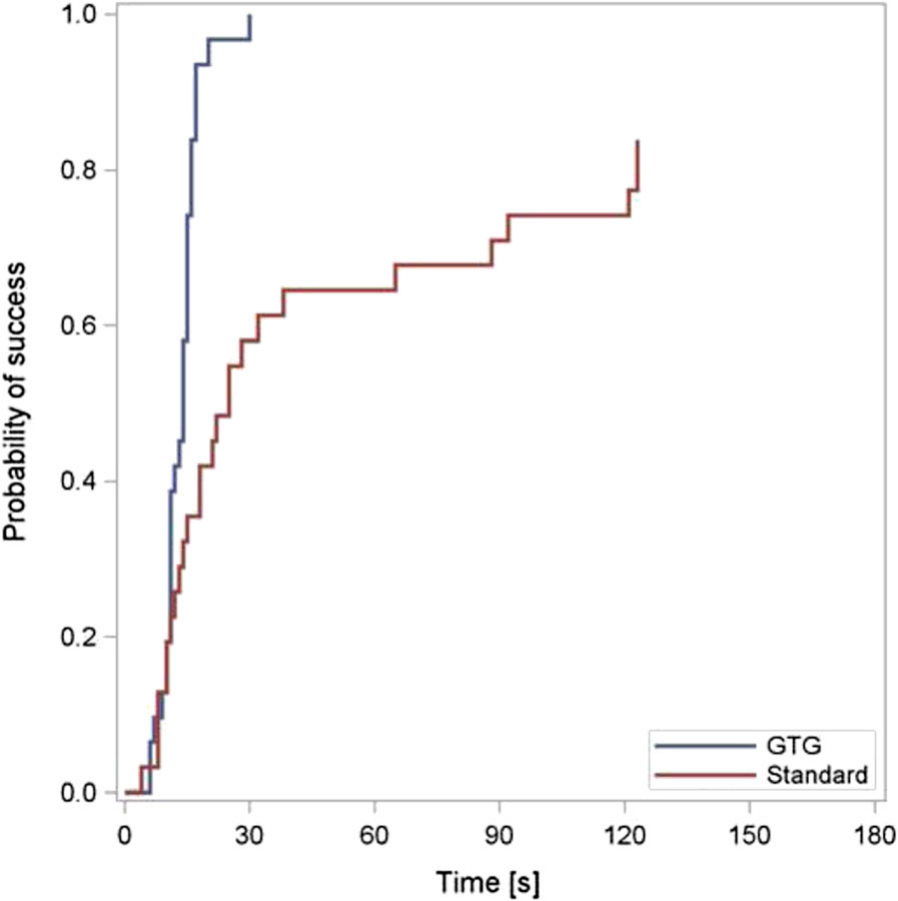
Probability of success depending on the required time – All participants were able to place the gastric tube with the GTG, in contrast to 26 out of 31 without the device (standard). The median time required for successful orogastric intubation was 25 (95% CI [14; 65]) seconds without and 14 (95% CI [11; 15]) seconds with the GTG [HR =5.4, *p* < 0.0001]

Twenty six participants (84%) were able to place the orogastric tube within 180 s without using the gastric tube guide. All participants (100%) were able to place the tube within 180 s when using the gastric tube guide (*p* = 0.0253). The median time required for orogastric intubation was 25 (95% CI [14; 65]) seconds without and 14 (95% CI [11; 15]) seconds with usage of the gastric tube guide, ranging from 4 to 123 s among those who successfully placed the gastric tube without the device and 6 to 20 s with the guide (Fig. 3). In the proportional hazard regression, we found no significant period effect (HR = 0.72, *p* = 0.2522 for period 1 vs period 2) but a significant treatment effect (HR =5.4, *p* < 0.0001 for gastric tube guide vs standard method). Table 1.

Of the participants, 22 (71%) needed only one attempt to place the gastric tube without the device, five (16%) made two attempts, four of which were successful and four (13%) made three attempts – all without success. In contrast, 30 (97%) of the participant were successful at the first attempt with the guide and only one participant (3%) needed a second attempt to successfully place the gastric tube (*p* = 0.0293). All participants were able to remove the gastric tube guide without removing the orogastric tube. There was no malpositioning of the orogastric tube in the trachea in both groups. When rating the two methods, 26 (84%) of the participants preferred the gastric tube guide to the standard method, whereas only one (3%) preferred the standard method to the gastric tube guide and four (13%) rated both methods in the same category (*p* = 0.0499). Table 2.

**Table 1:** Results

		Standard - STD			GTG			
Participant	Training	Time [s]	Attempts [n]	Rating [1–6]	Time [s]	Attempts [n]	Rating [1–6]	Sequence
1	Specialist	38	1	4	16	1	2	STD, GTG
2	Specialist	123	2	4	12	1	2	STD, GTG
3	Nurse	22	1	3	16	1	1	GTG, STD
4	Nurse	fail	3	6	20	1	2	GTG, STD
5	Nurse	15	1	3	14	1	3	STD, GTG
6	Nurse	fail	3	5	14	1	2	STD, GTG
7	Resident	21	1	3	17	1	2	GTG, STD
8	Specialist	123	2	4	15	1	2	STD, GTG
9	Resident	25	1	3	15	1	1	GTG, STD
10	Resident	28	1	3	17	1	1	GTG, STD
11	Resident	88	1	4	15	1	1	GTG, STD
12	Resident	10	1	2	7	1	1	STD, GTG
13	Resident	14	1	2	11	1	1	GTG, STD
14	Nurse	121	2	3	11	1	1	STD, GTG
15	Nurse	25	1	3	14	1	1	GTG, STD
16	Nurse	fail	3	5	15	2	2	GTG, STD
17	Specialist	8	1	1	11	1	1	STD, GTG
18	Nurse	11	1	3	11	1	3	STD, GTG
19	Specialist	32	2	6	10	1	1	STD, GTG
20	Resident	fail	3	6	17	1	1	STD, GTG
21	Specialist	4	1	2	6	1	3	GTG, STD
22	Nurse	65	1	3	14	1	1	STD, GTG
23	Nurse	8	1	1	13	1	1	GTG, STD
24	Resident	fail	2	5	30	1	1	GTG, STD
25	Nurse	18	1	4	11	1	2	STD, GTG
26	Resident	8	1	3	6	1	1	STD, GTG
27	Nurse	10	1	2	10	1	1	STD, GTG
28	Nurse	18	1	2	15	1	1	GTG, STD
29	Specialist	13	1	4	9	1	2	STD, GTG
30	Nurse	12	1	4	11	1	1	STD, GTG
31	Resident	92	1	4	16	1	1	GTG, STD

**Table 2:** Summary

	Standard	GTG	p
Median placement time and range (s)	25 [4–123]	14 [6–20]	<0.0001
Overall success	26/31 (84%)	31/31 (100%)	0.0253
Prefered method	1/31 (3%)	26/31 (84%)	0.0499

## Discussion

In our study we showed that using the gastric tube guide to place orogastric tubes leads to a higher success rate and a significantly shorter placement-time in manikins. In addition most users preferred the procedure when using the tool compared to the procedure without the gastric tube guide.

Usually gastric tubes will be placed using the oral cavity when they will be removed immediately after the procedure or before the patient recovers from anaesthesia. Compared to gastric tubes inserted through the nostril these techniques will most likely reduce complications like epistaxis, rhinitis and sinusitis. Other than that complications like laryngeal injuries, misplacement of the gastric tube and esophageal perforation might be the same for naso- and orogastric tubes [8]. As the placement of a gastric tube is a common procedure and has a high first-time failure rate various techniques have been described that should ease the procedure. Not considering laryngoscopy, these can be divided into two groups: the first group is characterized by movements of the head and neck to avoid the tip of the gastric tube to hit the common sites of resistance at the laryngeal level, e.g., applying lateral neck pressure or forced head flexion; the second group uses various methods to increase the stiffness of the gastric tube, e.g., freezing the tube or usage of a guide-wire [3]. A relatively new technique uses a slit endotracheal tube as a container to place the much softer gastric tube without the risk of kinking or knotting [9]. This procedure is similar to the placement of gastric tubes through supraglottic airway devices with a separate channel for esophageal access that have emerged during the last years [10]. After blind placement of the device a gastric tube can easily be placed through the second opening [11]. However, malpositioning of supraglottic airway devices is also known: Studies report incorrect positioning during in-hospital and prehospital conditions [12, 13].

The common sites of resistance for the tip of the gastric tube are the arytenoid cartilages and piriform sinuses [4], as the gastric tube guide most likely will keep the gastric tube away from these sites this may be a reason for the faster placement of the orogastric tube when using the gastric tube guide. This may as well be due to the increased stiffness of the material used for the guide compared with typical orogastric tubes. A study using slit tracheal tubes for placement of gastric tubes showed an increased risk for mucosal bleeding during this procedure. They suggest using softer, round and thinner material for the guide than used for tracheal tubes [9], a requirement that is fulfilled by the gastric tube guide accordingly to the manufacturer.

It is recommended to insert a gastric tube under direct laryngoscopic vision in sedated or anaesthetised patients. This is especially true for high-risk patients who are on antiplatelet or antithrombotic medications as they have a greater likelihood of bleeding [5]. In addition, laryngoscopies may also result in injuries to the tongue and teeth. Furthermore, blood in the oropharynx can be the result of traumatic insertion of the gastric tube, particularly if the gastric tube was inserted without actively creating sufficient retropharyngeal space by means of a chin lift manoeuver or with a laryngoscope. After correct placement of the gastric tube guide as a container, a gastric tube, advanced through the device, should reach the stomach easily if there are no pathological findings. Another problem of inserting a gastric tube under direct or indirect laryngoscopy is the space in the oral cavity: The oral tube and the blade often do not allow placing the gastric tube – although the esophageal entrance is under direct or indirect vision.

Our findings showed a significant reduction of the time needed for successful orogastric intubation from 25 (95% CI [14; 65]) seconds without to 14 (95% CI [11; 15]) seconds with the gastric tube guide. Although the reduction of 11 s may not be relevant in clinical routine we suppose that this effect will be greater in the clinical setting as the placement time in general was short during our study. Another important finding was the reduction of attempts until an orogastric tube could be inserted when using the gastric tube guide as every attempt increases the risk of complications especially for mucosal bleeding and laryngeal injuries.

A limitation of our study may be the applied time limit of 180 s. This was done for two reasons: First, we wanted to reduce the effect of personal motivation on the attempts performed, especially on when to stop the procedure in case of several failed attempts. Second, as the placement of orogastric tubes can be time consuming we wanted a defined cut-off. Indeed no participant was able to place an orogastric tube after 123 s which might be due to reduced stiffness of the tube after warming up [2], so the time limit did not alter the results of the overall success rate.

Manikin studies in general have been proven to be a reliable surrogate for clinical scenarios. On one hand the setting cannot simulate the precise conditions in a real patient in this context especially the warming to body temperature and the presence of various body fluids, on the other hand the use of manikins allows a strict standardization of study conditions. For that reason further clinical studies are necessary to determine the benefits and risks when using the gastric tube guide to place orogastric tubes.

## Conclusion

Our study showed that the usage of the gastric tube guide to place orogastric tubes in a simulation manikin leads to a higher success rate and a faster placement time and may therefore also be beneficial in clinical routine to reduce complications associated with the placement of orogastric tubes.

## Abbreviations

Fr: French
GTG: Gastric tube guide
